# Chrononutrition in Critical Illness

**DOI:** 10.1093/nutrit/nuae078

**Published:** 2024-06-21

**Authors:** Eylul Sagun, Asli Akyol, Cetin Kaymak

**Affiliations:** Faculty of Health Sciences, Department of Nutrition and Dietetics, Hacettepe University, Ankara, 06100, Turkey; Faculty of Health Sciences, Department of Nutrition and Dietetics, Hacettepe University, Ankara, 06100, Turkey; Gülhane Faculty of Medicine, Department of Anesthesiology and Reanimation, University of Health Sciences, Ankara Training and Research Hospital, Intensive Care Unit, Ankara, 06230, Turkey

**Keywords:** circadian rhythm, chrononutrition, intermittent feeding, critical illness

## Abstract

Circadian rhythms in humans are biological rhythms that regulate various physiological processes within a 24-hour time frame. Critical illness can disrupt the circadian rhythm, as can environmental and clinical factors, including altered light exposure, organ replacement therapies, disrupted sleep–wake cycles, noise, continuous enteral feeding, immobility, and therapeutic interventions. Nonpharmacological interventions, controlling the ICU environment, and pharmacological treatments are among the treatment strategies for circadian disruption. Nutrition establishes biological rhythms in metabolically active peripheral tissues and organs through appropriate synchronization with endocrine signals. Therefore, adhering to a feeding schedule based on the biological clock, a concept known as “chrononutrition,” appears to be vitally important for regulating peripheral clocks. Chrononutritional approaches, such as intermittent enteral feeding that includes overnight fasting and consideration of macronutrient composition in enteral solutions, could potentially restore circadian health by resetting peripheral clocks. However, due to the lack of evidence, further studies on the effect of chrononutrition on clinical outcomes in critical illness are needed. The purpose of this review was to discuss the role of chrononutrition in regulating biological rhythms in critical illness, and its impact on clinical outcomes.

## INTRODUCTION

During the evolution of life on Earth, the need for most organisms to adapt to the cycle of the sunrise and sunset led to the development of biological rhythms. The development of biological rhythms as an internal timekeeping system is believed to be a survival strategy in response to constantly changing environmental factors.^1^ Circadian rhythms (circa=around and *dies*=1 day) in humans are biological rhythms that regulate various physiological processes within a 24-hour time frame. The circadian system is regulated by tissue-specific clocks controlled by the suprachiasmatic nucleus (SCN) of the hypothalamus.^2^ In order for the circadian system to synchronize with the environment, external time cues called zeitgebers need to be perceived. The biological clock primarily synchronizes with the light/dark cycle. Light as a photic zeitgeber is one of the major zeitgebers for humans. Food, exercise, temperature, and sounds are nonphotic zeitgebers, and together all these external cues elicit appropriate biological responses in the body.^3^ When light reaches the eye, the light stimulus is primarily detected by retinal ganglion cells that convert photon energy into an electrical signal.^4^ This signal is later relayed directly to the SCN and other target regions in the brain. As a result, metabolic, neural, and humoral signals are generated, serving as messengers to synchronize peripheral clocks in the body to achieve an appropriate rhythmic organization of physiological processes.^5^ At the molecular level, circadian rhythms are regulated by transcription factors and cellular proteins providing autoregulatory transcription–translation feedback loops.^6^ More specifically, 2 master transcription factors that generate oscillating transcriptional rhythms are involved in the daily regulation of physiological processes: circadian locomotor output cycles kaput (CLOCK) and brain and muscle aryl hydrocarbon receptor nuclear translocator (BMAL1). CLOCK and BMAL1 create a heterodimeric transcription factor that binds to enhancer (E)-box elements of target genes, including period circadian regulators (PER) 1/2/3 and cryptochrome (CRY) 1/2, and elicits their expression. Hereby PER and CRY proteins accumulate in the cytoplasm, forming the heterodimer complex that is translocated to the nucleus to downregulate its own expression by inhibiting CLOCK/BMAL1-mediated transcription.^7^ In addition, this transcriptional network modulates the expression of other genes found in various tissues. These clock-controlled genes are indispensable for cell metabolism.^8^

In recent years, many factors have been associated with the disruption of circadian rhythms. Critical illness in itself can disrupt the circadian rhythm, and the 24-hour care provided in the intensive care unit (ICU) is also likely to undermine the biological rhythm. Misalignment of the biological rhythms are likely to interfere with a patient’s critical condition.^9^ Recently, a new field called ‘chrononutrition’ has emerged, which explores the relationship between nutrition and circadian rhythm.^10^ Understanding this interaction and developing strategies to prevent circadian misalignment in the ICU might improve patient recovery. The present review aimed to discuss the role of chrononutrition in regulating biological rhythms in critical illness and affecting clinical outcome.

## METHODS

The literature review was carried out by searching the following keywords: “circadian rhythm,” “clock genes,” “chronodisruption,” “circadian desynchronization,” “critical illness,” “intensive care,” “sleep,” “nutrition,” “chrononutrition,” “enteral nutrition,” “metabolism,” “meal timing,” “time-restricted feeding,” “intermittent feeding,” “dietary pattern,” “peripheral clock,” “children,” and “fasting” in the databases “PubMed, ScienceDirect, Google Scholar, Web of Science, and Scopus” through Hacettepe University Library. Selected articles published prior to September 2023 were included in the present study. Randomized controlled trials, cohort studies, cross-sectional studies, experimental animal intervention studies, narrative reviews, systematic reviews, and meta-analyses were listed as eligible study designs for inclusion.

## CAUSES OF CIRCADIAN MISALIGNMENT IN THE ICU

**Figure 1.**
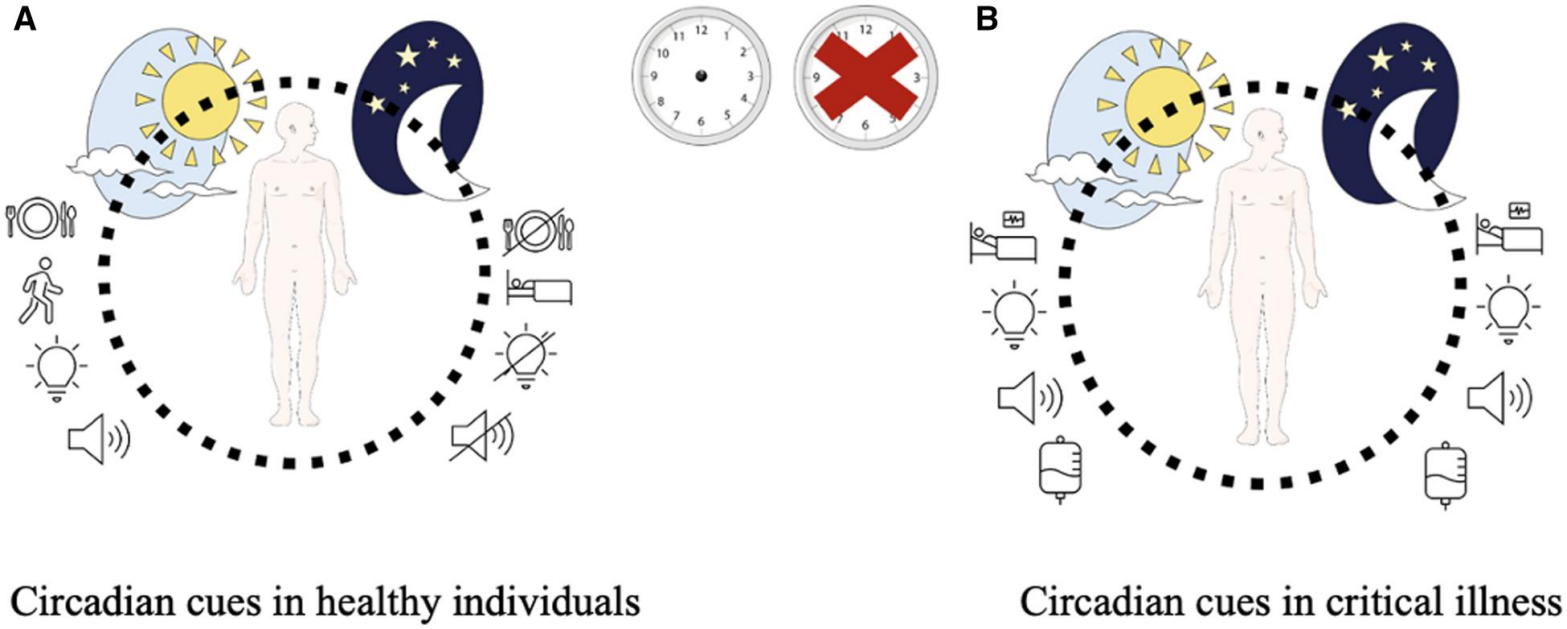
Comparison of Circadian Cues in Healthy Individuals and Critical Illness. Altered light exposure, noise, continuous feeding schedule, immobility and therapeutic interventions are the causes of circadian misalignment in ICU (right panel). The figure was partly generated using Servier Medical Art, provided by Servier, licensed under a Creative Commons Attribution 3.0 unported license

Potential factors contributing to the disruption of circadian rhythms in critically ill patients include altered light exposure, organ replacement therapies, disrupted sleep–wake cycles, noise, continuous feeding, immobility, and therapeutic interventions (Figure 1).^9^^,^^11^ Light serves as the primary zeitgeber, and the lack of typical light intensity and exposure to light during inappropriate hours could be the primary factor behind the abnormal rhythms observed in patients within the ICU. Similarly, patient care interactions such as medication administration, checking blood pressures, phlebotomy, and wound care, and noise sources, including conversations among healthcare staff and the sound of the patient care equipment, are considered to be circadian rhythm disruptors.^12^ It is reported that there are similar noise levels between night and day.^13^ Furthermore, this affects the sleep quality of the patients and disrupts their circadian rhythm.^13^ Sedative medications can alter sleep–wake patterns, and corticosteroids can interfere with the natural cortisol release rhythm. Disruption of this system may have significant physiological consequences, potentially leading to a dysregulated circadian clock.^12^^,^^14^ Last but not least, feeding pattern is another significant cause for circadian misalignment. Nutrition should exhibit proper synchronization with SCN-driven endocrine signals, as food intake establishes rhythms that need to be kept in sync with the central clock in metabolically active peripheral tissues and organs.^15^^,^^16^ Feeding patterns that do not align with the biological rhythm can further disrupt the circadian rhythm and lead to other physiological issues.^17^ Indeed, recent studies showed circadian misalignment in ICU. These studies will be discussed in the following section.

### Circadian misalignment in the ICU: evidence from the literature

There is significant evidence indicating that critically ill patients monitored in ICU exhibit circadian rhythm disturbances (Table 1).^18–23^ As it is not feasible to directly measure circadian rhythm in humans, circadian rhythm can be evaluated through measurements of core body temperature, melatonin levels, cortisol levels, or the expression of circadian clock genes.^12^ Gazendam et al^18^ studied core body temperatures over a 48-hour period to investigate circadian rhythms in patients in the ICU. Their findings suggested that circadian rhythms were present but altered in patients in the ICU. Their lowest value of core body temperature was found to be distributed over a 24-hour period, unlike that of healthy individuals, which occurs in the early morning. Additionally, a significant correlation has been found between circadian rhythm disturbances and the severity of critical illness, as indicated by the Acute Physiology and Chronic Health Evaluation (APACHE) III score.^18^ In another study, investigators aimed to assess and compare the immune circadian rhythms in trauma patients with or without the development of sepsis. They found that the circadian rhythms of cortisol, cytokines, leukocytes, BMAL1 expression, PER2 expression, and PER3 expression were all disrupted in trauma patients. Early disruption of circadian rhythms was associated with the development of sepsis and could be an indicator of the severity of sepsis.^19^ Beyer et al^20^ conducted cosinor analysis, using hourly blood pressure measurements from patients in the eICU Collaborative Research Database. They calculated the amplitude of the 24-hour circadian rhythm and the time of day when blood pressure peaked. They found that a higher APACHE-IV score, sepsis, organ dysfunction, and mechanical ventilation were associated with a lower-amplitude and disrupted circadian rhythm.^20^ In a cross-sectional study, 15 intensive care patients and 11 healthy participants were evaluated. Through the analysis of blood samples taken every 2 hours, commencing within a day after admission to the emergency department, it was observed that rhythmic expression of *BMAL1, CRY1*, and *PER1* was not present in the patients, while rhythmic expression of circadian genes was observed in healthy controls. According to the authors, gene expression rhythms become abnormal during critical illness.^21^ Diaz et al^22^ conducted a study with 11 patients in the neurology ICU. In their research, they assessed the rhythm *CLOCK*, *BMAL1*, *CRY1*, and *PER2* gene expressions on the first day and 1 week after admission to the ICU. Their results showed that the rhythmic expression of the four genes had completely disappeared 1 week later.^22^ In a retrospective observational study, 29 448 patients’ data from the eICU Collaborative Research Database were used to determine the circadian rhythms of vital signs and establish whether there was any association with in-hospital mortality. There were significant differences in the circadian rhythms of heart rate, respiration rate, and pulse oximetry–derived oxygen saturation between survivors and non-survivors.^23^ As observed in studies published in recent years, circadian rhythm disruption has been identified and linked to morbidity and mortality in critical illness. However, the studies conducted exhibit limitations due to variations in patient populations and differences in methods of assessing circadian rhythm.

**Table 1.** Studies that have Evaluated Circadian Misalignment in ICU

Study	Sample size	Study design	Methodology	Main findings
Gazendam et al (2013)^18^	*n* = 21 ICU patients (59 ± 11 years of age; 8 men and 13 women)	Observational study	Core body temperature (CBT) recordings were made for 48 hours at a rate of one sample every 5 min.	Circadian rhythms of CBT were present but altered in patients. A significant correlation has been found between circadian rhythm disturbances and APACHE-III scores.
Coiffard et al (2019)^19^	*n* = 38 severe trauma patients (13 septic and 25 non-septic patients)	Prospective observational study	Blood samples were collected from patients within 4 days post admission, with collections taking place every 4 hours over a 24-hour period. From blood samples, cortisol and cytokines (IL-6, IL-10, and TNF-α) were measured with immunoassays. BMAL1, PER2 and PER3 mRNA expression levels were analyzed. Neutrophils, monocytes, and lymphocytes were analyzed by flow cytometry.	Circadian rhythms of cortisol, cytokines; leukocytes; and the expression of BMAL1, PER2, and PER3 were all disrupted in trauma patients. Early disruption of circadian rhythms was associated with the development of sepsis and could be an indicator of the severity of sepsis.
Beyer et al (2021)^20^	*n* = 23 355 patients from the recorded eICU Collaborative Research Database	Retrospective observational study	Circadian variation was analyzed by fitting cosinor models to hourly blood pressure (BP) measurements in patients registered in the eICU Collaborative Research Database, with an ICU length of stay of at least 3 days. The amplitude of the 24-h circadian rhythm and time of the day when BP peaked were determined.	A higher APACHE-IV score, sepsis, organ dysfunction, and mechanical ventilation were associated with a lower-amplitude and disrupted circadian rhythm.
Maas et al (2020)^21^	*n* = 15 ICU patients (10 septic patients) and 11 healthy volunteers	Cross-sectional observational study	mRNA expression levels of CRY1, CRY2, PER1, PER3, BMAL1, and CLOCK were evaluated by blood samples taken every 2 hours within a day from admission to the emergency department.	BMAL1, CRY1 and PER1 rhythmic expression was not present in the patients, whereas rhythmic expression of circadian genes was observed in healthy controls.
Diaz et al (2020)^22^	*n* = 11 neuro-ICU patients	Prospective observational study	The rhythm of *CLOCK*, *BMAL1*, *CRY1*, and *PER2* genes in patients on the first day after admission in the ICU and 1 week later was studied, at 4 time points throughout the day: at 6, 12, 18, and 24 hours.	mRNA expression for the 4 clock genes was shown to have rhythmicity on the first day after admission to the ICU. After 1 week, the clock gene rhythmicity had completely disappeared.
Yang et al (2023)^23^	*n* = 29 448 critically ill patients from the recorded eICU Collaborative Research Database	Retrospective observational study	The circadian rhythms of vital signs (heart rate [HR], temperature, respiration rate [RR], pulse oximetry–derived oxygen saturation [SpO_2_], and blood pressure [BP]) were analyzed in critically ill patients using the cosinor method. In-hospital mortality and APACHE-IV scores were calculated.	There were significant differences in the circadian rhythms of heart rate, respiration rate, and pulse oximetry–derived oxygen saturation between survivors and non-survivors. Mesor, amplitude, and peak time of HR, RR, and SpO_2_ combined with the APACHE IV score were shown to be good indicators for in-hospital mortality.

### How to treat circadian misalignment in ICU: regulation of central/peripheral rhythms

Interventions aimed at enhancing circadian function in the ICU are inclined to be complex, given the multifaceted nature of circadian disruption and the diverse array of implicit and identified risk factors, involving multiple components. Nonpharmacological interventions, controlling the ICU environment, and pharmacological treatments, are among the treatment strategies for circadian disruption.^11^ To restore circadian misalignment, the primary emphasis appears to be on enhancing the quality of sleep. According to the American Thoracic Society, key components of sleep promotion interventions include optimizing zeitgebers, ensuring patient comfort, treating preexisting sleep disorders, controlling the environment, adjusting medication dosage, and employing a multidisciplinary approach.^11^ Multiple factors such as anxiety, pain, preexisting sleep disorders and ICU environment attenuate sleep quality.^24^ Therefore, the implementation of various relaxation techniques (such as music therapy), the optimization of ICU environments (by adjusting room temperature, lighting, and noise levels), and the provision of sleep bundles, eye masks, or earplugs appear promising as nonpharmacological approaches to enhancing sleep quality.^25–27^ Up to date, there are no ICU guideline recommendations endorsing the utilization of pharmacological treatment to alleviate circadian disruption in ICU.^28^ Nevertheless, medications such as melatonin, ramelteon, and quetiapine are prescribed to improve sleep quality in ICU.^29^ Some of these pharmacological treatments have been associated with promising results,^30^^,^^31^ while others have not.^32^ It is important to note that, when appraising interventions aimed at enhancing sleep quality, limitations become evident. These limitations arise from small sample sizes, the exclusion of mechanically ventilated patients with severe illness, and the reliance on patient-reported sleep quality.^33^

Utilizing circadian cues as treatment strategies holds significance in circadian regulation. For example, light therapy emerges as a crucial approach for optimizing photic zeitgebers. Interventions involving bright daytime light and minimal or no light exposure during the night play a pivotal role in the regulation of central rhythms.^34^ Given that light signals stimulate the SCN in the brain and synchronize peripheral clocks in the body, ensuring proper light exposure becomes imperative for the appropriate rhythmic organization of physiological processes.^4^^,^^5^ In a randomized controlled pilot study, appropriately timed light therapy has been demonstrated as normalizing circadian rhythms in critically ill patients.^35^ According to a systematic review, light therapy in hospitalized patients seems to be beneficial, but due to the small sample sizes there was insufficient data to justify recommending an intervention.^36^ Peripheral clocks are known to be entrained by nonphotic zeitgebers such as feeding schedules and exercise. Under normal conditions, diet establishes biological rhythms in metabolically active peripheral tissues and organs through appropriate synchronization with endocrine signals regulated by the SCN.^37^ Indeed, feeding time plays a crucial role as a circadian cue. It particularly influences peripheral clocks in the gut, liver, and pancreas.^38^ Disruption of the interplay between nutrient availability and the circadian clock results in the dysregulation of metabolic processes intricately associated with endocrine signals modulated by dietary inputs.^39^ In animals, feeding during the inactive phase induces a complete reversal in the expression of core clock genes within skeletal muscles, adipose tissue, and the liver.^40^ Consequently, scheduling mealtimes is recommended to align with biological rhythms to preserve optimal circadian health. Misalignment of peripheral clocks is linked to circadian disruption, insulin resistance, cardiovascular dysfunction, and impaired immune response.^41–44^ Therefore, adhering to a feeding schedule based on the biological clock, a concept known as “chrononutrition,” appears to be vitally important for regulating peripheral clocks.^45^

## CHRONONUTRITION

“Chrononutrition” involves examining the interplay between biological rhythms and nutrition, as well as exploring the correlation between these elements and human health.^46^ It is a dietary approach that aligns with our biological clock, aiming to take account of variations in metabolism throughout the day.^47^ Chrononutrition involves the timing, frequency, and regularity of meals and the relative significance of these factors in terms of metabolic health.^48^

Eating behavior is regulated by the energy requirements of the organism. The necessity to supply a consistent amount of energy to tissues is a homeostatic drive that adapts feeding behavior to the energetic state of the organism. Food intake also exhibits a circadian variation synchronized with the light–dark cycle and food availability; for instance, mice typically consume more than 70% of their food during the dark/active phase of the day.^49^ The significance of meal timing is linked to our circadian clock and its influential role in regulating metabolic processes throughout the body. The molecular components constituting the circadian clock are highly responsive to the energy state of the organism, food intake, and related cellular signals.^50^

**Figure 2.**
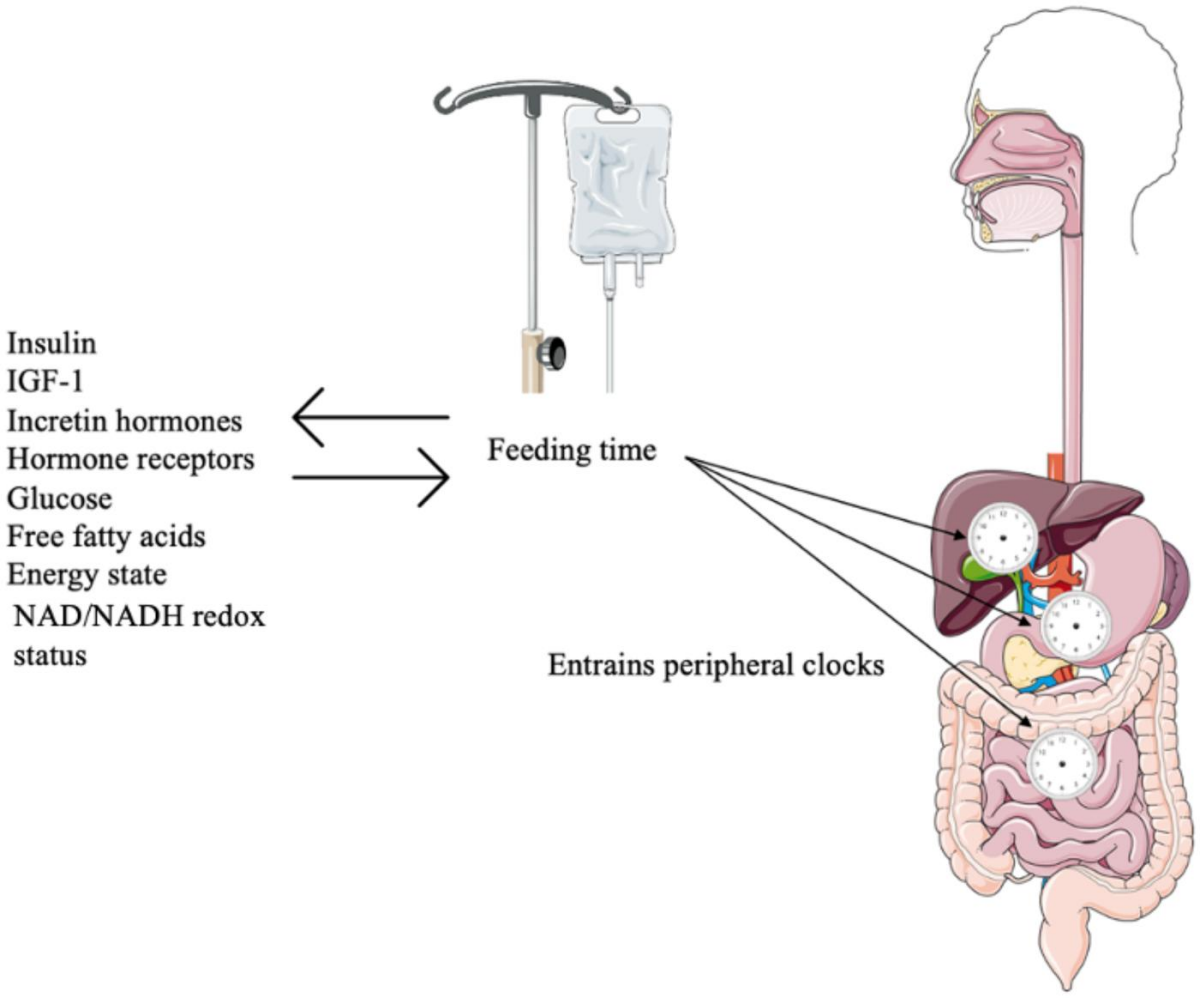
Nutrition Establishes Biological Rhythms in Metabolically Active Peripheral Tissues and Organs Through Appropriate Synchronization With Endocrine Signals. Energy state, cellular NAD/NADH redox status, hormone receptors and hormonal regulators, particularly insulin and glucagon, interact with the clock genes in the peripheral tissues such as liver and adipose tissue. The figure was partly generated using Servier Medical Art, provided by Servier, licensed under a Creative Commons Attribution 3.0 unported license. *Abbreviations*: IGF-1, insulin-like growth factor-1; NAD, nicotinamide adenine dinucleotide; NADH, reduced nicotinamide adenine dinucleotide

Hormone receptors (peroxisome proliferator activated receptor [PPAR]α and PPARγ), circadian gene interactions in liver and adipose tissue, and intracellular oxidation/reduction (redox) reactions collectively regulate energy metabolism at the cellular level. These pathways significantly interact with the clock genes in the peripheral tissues.^51^ For example, PPARα, a member of the nuclear hormone receptor family, activates ketogenesis and hepatic fatty acid oxidation in response to starvation. Its direct binding to the promoter region of the BMAL1 protein establishes a regulatory loop in which PPARα expression is modulated through the CLOCK:BMAL1 heterodimer.^52^^,^^53^ CLOCK proteins demonstrate efficient binding capabilities to E-box elements of target genes exclusively when there is the presence of reduced nicotinamide adenine dinucleotide (NADH) and nicotinamide adenine dinucleotide phosphate (NADPH). Conversely, the binding of the CLOCK:BMAL1 complex to DNA is hindered by oxidized NAD^+^ and NADP^+^ abundance. NAD^+^-dependent deacetylase sirtuin 1 (SIRT1) regulates CLOCK-mediated chromatin remodeling and the degradation of PER2 protein in a circadian manner.^54^ Therefore, the cellular NAD/NADH redox status can induce circadian phase changes by impacting the transcriptional activity of the *BMAL1* and *CLOCK* genes.^55^ Considering all this, it becomes evident that these nutritional pathways play a crucial role in the biological clock of the organism (Figure 2).^50–55^

## REGULATION OF PERIPHERAL RHYTHMS BY CHRONONUTRITION IN ICU

### Intermittent feeding

The timing of feeding is a predominant factor in determining circadian phase, especially in the peripheral clocks.^56^ When food access is limited to the usual resting phase of an organism, which is the night for humans or daytime for nocturnal rodents, peripheral clocks become decoupled from the SCN and adjust to the timing of food availability. Damiola et al^57^ demonstrated that time-restricted feeding under light–dark or dark–dark conditions alters circadian gene expression in peripheral cell types without inducing changes in gene expression in the SCN.^57^ Likewise, other studies with rodents have indicated that time-restricted feeding during the inactive phase significantly shifts the phase of circadian gene expression in peripheral tissues, while having no impact on the central clock, which is primarily regulated by the light/dark cycle.^58^^,^^59^ Another study shows that the entrainment of the liver clock induced by food depends on both the volume of food and the duration of fasting between 2 meals.^60^ It is asserted that food intake, particularly after prolonged fasting, is a potent factor in resetting peripheral clocks.^61^ In accordance with the feeding schedule, nutritional hormones secreted from the gastrointestinal tract in response to nutrient availability entrain peripheral gene rhythms.^55^

During the fasting and refeeding phases, crucial hormonal regulators, particularly glucagon and insulin, play a pivotal role in orchestrating the expression of *BMAL1* in the liver. In the fasting period, the activation of cAMP-response element-binding protein (CREB) and its coactivator CREB-regulated transcriptional coactivator 2 (CRTC2) by glucagon leads to their recruitment to the *BMAL1* promoter as a transcriptional complex, inducing *BMAL1* expression.^62^ Refeeding subsequent to fasting triggers insulin secretion, initiating the S42 phosphorylation of BMAL1 through the Akt pathway. This phosphorylation at S42 prompts the dissociation of BMAL1 from the E-box, thereby regulating the circadian expression of genes related to metabolism in hepatic cells.^63^ For instance, Crosby et al^64^ showed that elevated levels of insulin and insulin-like growth factor-1 (IGF-1) reset circadian clocks by inducing PER2 proteins, and misaligned insulin signaling disrupts the circadian organization of clock gene expression. This research suggests that insulin can function as a post-prandial signal, conveying meal timing information to circadian clocks throughout the body.^64^ A rodent study concluded that food intake resets liver transcription rhythms by inducing the core clock genes *PER1* and *PER2* through the stimulation of an anorexigenic incretin hormone oxyntomodulin (OXM) secretion from the intestine.^65^ Consequently, adjusting feeding time through biological rhythm seems to be undeniably important for metabolism. However, feeding during the resting phase instead of active phase induces a condition of internal circadian misalignment, which has been proposed as a potential cause for metabolic dysregulation.^66^

In critical illness, nutritional support can be provided either through enteral nutrition (EN) or parenteral nutrition (PN), based on the functionality of gastrointestinal system. Enteral nutrition is characterized by the delivery of nutrients to the gastrointestinal system through a tube, catheter, or stoma. Enteral nutrition administration methods include continuous, cyclic, intermittent, and bolus techniques.^67^ For critically ill patients, the optimal enteral feeding schedule is a matter of debate. Current guidelines recommend continuous infusion for these patients. However, it is indicated that in determining the most appropriate way for administration, the clinical and functional status of the patient should be considered, but emphasized that there may be insufficient available data to form a conclusive judgment on this matter.^68–70^ Continuous feeding delivers nutrition through an enteral feeding pump continuously over a 24-hour period. It is typically initiated at a rate of 20–50 mL/h and advanced to the goal rate with increments of 10–25 mL/hour every 4–24 hours. The intermittent enteral feeding method involves administering enteral products to the patient 4–6 times a day, in volumes ranging from 240 to 720 mL, over a period of 20–60 minutes, either through an infusion pump or gravity drip method.^71^^,^^72^ Continuous feeding offers advantages such as potential improvement in gastric tolerance and a decreased risk of aspiration. On the other hand, the intermittent enteral feeding method seems to be more physiological. However, it comes with increased risks of aspiration, potential glucose variability, and, in some cases, may contribute to delayed gastric emptying, resulting in an elevated gastric residual volume.^73^ According to a meta-analysis, continuous feeding is associated with lower risks of feeding intolerances and aspiration, whereas intermittent feeding is linked to a decreased incidence of constipation and increased calorie intake.^74^

In terms of glycemic control, Ren et al^75^ found no significant difference between continuous and intermittent fasting. In addition, intermittent feeding was shown to be as safe and tolerable as continuous feeding for critically ill patients.^75^ In another study, Sjulin et al^76^ demonstrated that critically ill patients requiring insulin infusion showed a decrease in insulin requirements when intermittently fed. However, the authors noted the need for larger-scale clinical trials to further investigate this effect.^76^ In a pilot study, the metabolic effects of a 12-hour feeding/12-hour fasting cycle were tested in 70 critically ill patients, revealing that a 12-hour fasting period significantly reduced insulin requirements and serum IGF-1 concentration.^77^ Insulin and IGF-1 have been recently acknowledged as circadian entrainers. Consequently, they can function as a fundamental signal indicating the time for cellular clocks to align with feeding schedules throughout the body.^64^ The established diurnal fluctuation in glucose tolerance is delineated by augmented glucose tolerance during the early part of the day, juxtaposed with attenuated responses as the evening transpires.^48^ Incretin hormones, notably glucagon-like peptide-1 (GLP-1) and glucose-dependent insulinotropic polypeptide (GIP), display diurnal variation, peaking in the early part of the day. This leads to more expeditious insulin responses to nutrient intake in the morning.^78^ Therefore, meal timing seems to be of vital importance for regulating peripheral rhythms. It is evident that more research is needed, to investigate the effects of feeding schedules on diurnal variation in glycemic control for critically ill patients. The consumption of nutrients during the biological night has been demonstrated to correlate with compromised insulin sensitivity and heightened insulin secretion from pancreatic beta-cells.^79^ Therefore 24-hour continuous feeding might be an important reason for peripheral circadian misalignment. Intermittent feeding including overnight fasting could restore this misalignment by resetting peripheral rhythms.^80^

To date, the relationship between intermittent feeding and circadian rhythm has never been investigated in intensive care. However, there is a limited amount of research about time-restricted feeding, metabolic health, and circadian health. These studies were primarily conducted with healthy or overweight/obese individuals and people with type 2 diabetes. Research on time-restricted feeding and metabolic health typically restricted daily energy intake to a window of 4–12 hours. For instance, in a study where dinner was shifted 6 hours earlier, participants exhibited enhancements in insulin sensitivity, blood pressure, and oxidative stress during the 5-week intervention, despite consuming the same amount of energy.^81^ In a different study, healthy men who practiced nighttime food restriction refrained from consuming calories between 19:00 and 06:00 daily for a period of 2 weeks. The findings revealed that the group adhering to nighttime food restriction consumed fewer daily calories, leading to a significant difference in weight change.^82^ Cienfuegos et al^83^ restricted energy intake in obese individuals for 8 weeks to either a 4-hour period (15:00–19:00) or a 6-hour period (13:00–19:00). In both interventions, they noted a decrease in body weight, insulin resistance, and oxidative stress.^83^ A randomized controlled study has indicated that time-restricted feeding (to 8 hours per day) did not alter core gene expression in muscle in overweight/obese men. However, it induced rhythmicity in various amino acid transporter genes and metabolites.^84^ In a study conducted with obese women, it was found that consuming a breakfast containing 30–35% of energy requirements before 8 am, followed by approximately 24 hours of fasting until 8 am the next day on three nonconsecutive days per week, was unable to synchronize peripheral clocks in adipose tissue and muscle tissue.^85^ Jakubowicz et al^86^ reported that the amplitude of *BMAL1, CRY1, PER2,* and* RORα* was higher in the white blood cells of individuals with type 2 diabetes who consumed three meals within a 12-hour period compared with those who had six meals within a 15-hour window.^86^ In light of all this information, it is evident that research on intermittent feeding should be extended to include critically ill patients.

### Nutrient composition

In addition to timing, macronutrient composition appears to be an important determinant for circadian health.^55^ Consumption of a balanced diet containing carbohydrates and proteins after fasting is beneficial for the entrainment of the peripheral circadian clock induced by restricted feeding during the inactive phase.^56^ Protein consumption is an important nutritional factor that regulates the circadian rhythm. Yokota et al demonstrated that under 12-hour light/12-hour dark conditions, mice fed with a low-protein diet for 7 days showed the elimination of the circadian rhythm of serum insulin and hepatic lipid metabolism.^87^ Another study evaluated the effects of a low-carbohydrate, high-protein diet on peripheral clocks in mice. After 2 weeks of intervention, expression levels of key gluconeogenic regulatory genes and *PPARα* had increased in the liver and kidneys. Additionally, although *PER2* expression had not increased, *BMAL1* and *CRY1* mRNA expressions were elevated in the liver and kidneys. This suggests that high protein consumption can adjust the molecular clocks in peripheral tissues.^88^ According to the ESPEN Guideline, during critical illness, 1.3 g/kg/day of protein equivalents can be delivered progressively.^68^ In addition to circadian regulation, high protein intake may show benefits when proteolysis and muscle loss are considered. However, due to the risk of overfeeding, optimal protein targets should be determined based on the patient’s needs.^68^ While carbohydrates are the preferred substrate for energy production in cases of critical illness, insulin resistance and hyperglycemia often occur as common secondary responses to stress.^89^ Providing an excess of energy through glucose is linked to hyperglycemia, heightened CO_2_ production, increased lipogenesis, and elevated insulin requirements.^90^ High-carbohydrate foods induce insulin secretion, subsequently influencing the post-transcriptional modulation of BMAL1 protein, and impacting the hepatic circadian clock.^63^ The use of fiber-rich diabetic-specific enteral formula in ICU patients with type 2 diabetes may improve the glycemic response.^68^ Furthermore, short-chain fatty acids, which are derived from gut microbiota as a result of soluble fiber fermentation, have been found to modify the peripheral clock in mice.^91^ Hence, incorporating enteral products containing fiber may contribute to enhancing glycemic responses and regulating peripheral clocks.

On the other hand, high-fat diets seem to disrupt biological rhythms. Kohsaka et al^92^ found that a chronic high-fat diet alters the phase of the circadian rhythm in the liver in mice. Also, short-term exposure to a high-fat diet was found to disrupt the expression of circadian rhythm and trigger inflammation and oxidative stress.^93^ However, the type of the fat has been found to be just as important as the amount of fat for circadian regulation. In a rodent trial, food containing fish oil or docosahexaenoic acid (DHA)/eicosapentaenoic acid (EPA) facilitated restricted-feeding–induced phase shifts of liver circadian gene expression and increased insulin secretion through GPR120 (a polyunsaturated fatty acid receptor) in mice.^94^ According to the ESPEN guideline, EN enriched with omega-3 fatty acids can be administered but should not be given by bolus administration or on a regular basis.^68^ Based on the findings of a meta-analysis, the use of enteral fish oil as a supplement for critically ill patients is not recommended, due to the lack of strong evidence supporting clinical benefits. Nevertheless, positive effects on mortality have been noted in patients with acute respiratory distress syndrome (ARDS). However, large-scale studies are needed to investigate the relationships between the administration of fish oil to critically ill patients, clinical outcomes, and circadian rhythm.^95^

The proficient metabolism of primary nutrients essential for protein synthesis and energy production necessitates a sufficient and balanced provision of all requisite trace elements and vitamins. Therefore, in critical illness, micronutrient provision and monitoring during nutritional support is important for metabolic health.^96^ By fostering improvements in metabolic health, micronutrients may indirectly contribute to the enhancement of circadian well-being. Nevertheless, there is a lack of data regarding the use of micronutrients for circadian health in critical illness. In addition, the effects of certain bioactive substances such as caffeine,^97^ cinnamic acid,^98^ L-theanine,^99^ nobiletin,^100^ and melatonin^101^ on circadian health have mainly been investigated in animal models. There is currently no recommendation regarding the use of these substances in intensive care.

## CHRONONUTRITION IN THE NEONATAL AND PEDIATRIC CRITICAL CARE

According to the current guidelines, EN should be initiated in critically ill term neonates and children, unless it is contraindicated.^102^^,^^103^ Early initiation of EN within the first 24–48 h after admission to a neonatal ICU (NICU) or a pediatric ICU (PICU) in eligible patients is recommended. However, due to the heterogeneity of the studies and the lack of high-quality evidence, the European Society of Paediatric and Neonatology Intensive Care indicates that neither continuous nor intermittent feeding is superior as a feeding method.^103^ There are no studies that have investigated intermittent feeding as a chrononutritional approach, and its effects on clinical outcomes. Limited studies have compared different feeding methods in NICU and PICU. Kumar et al^104^ compared continuous and intermittent tube feeding in critically ill children and showed no difference in the time taken to provide target calories and protein between the two different modes of delivery of EN. In a prospective cohort study, intermittent and continuous EN was compared in mechanically ventilated critically ill children, and feeding method was found not to be associated with differences in energy and protein intakes in the first 7 days of admission.^105^ Veldscholte et al^106^ aimed to investigate whether intermittent feeding coupled with overnight fasting, compared with 24-hour continuous feeding, led to an increased fasting response, characterized by heightened ketosis, and whether it was both feasible and safe in critically ill children. Accordingly, there was no significant difference in feeding intolerance or hypoglycemic event incidence between the 2 groups. However, during the first 4 days of PICU, lower nutrient intakes were observed in the intermittent feeding group.^106^ It is evident that additional randomized trials are needed in order to clarify the efficacy of intermittent feeding in relation to clinical and chronobiological outcomes.

## CONCLUSION

Circadian misalignment during critical illness interferes with the patient’s critical condition. Therefore, efforts to regulate this misalignment may lead to improvements in clinical outcomes. There are significant modifiable clinical and environmental circadian disruptors, including light, temperature, physical activity, noise, preexisting sleep disorders, stress, and medication. In addition, a 24-hour continuous feeding schedule might be a particularly important factor contributing to peripheral circadian misalignment. Hence, chrononutritional approaches such as intermittent enteral feeding that includes overnight fasting and consideration of macronutrient composition in enteral solutions could potentially restore circadian health, by resetting peripheral clocks. Further investigations are necessary to clarify the relationship between these interventions and possible outcomes.

## Data Availability

No new data were generated or analysed in support of this research.
